# Impact of cardiac catheterization timing and contrast media dose on acute kidney injury after cardiac surgery

**DOI:** 10.1186/s12872-018-0928-8

**Published:** 2018-10-05

**Authors:** Wuhua Jiang, Jiawei Yu, Jiarui Xu, Bo Shen, Yimei Wang, Zhe Luo, Chunsheng Wang, Xiaoqiang Ding, Jie Teng

**Affiliations:** 1 Department of Nephrology, Zhongshan Hospital, Shanghai Medical College, Fudan University, No 180 Fenglin Rd, Shanghai, 200032 China; 2 Shanghai Institute of Kidney and Dialysis, Shanghai, China; 3 Shanghai Medical Center of Kidney, Shanghai, China; 4 Department of Nephrology, Xiamen Branch, Zhongshan Hospital, Fudan University, Xiamen, China; 5 Shanghai Key Laboratory of Kidney and Blood Purification, Shanghai, China; 6 Department of Cardiac Surgery Intensive Care Unit, Zhongshan Hospital, Shanghai Medical College, Fudan University, Shanghai, China; 7 Department of Cardiovascular Surgery, Zhongshan Hospital, Shanghai, China

**Keywords:** Acute kidney injury, Cardiac surgery, Cardiac catheterization, Contrast media

## Abstract

**Background:**

The association between pre-operative cardiac catheterization and cardiac surgery associated acute kidney injury (CSA-AKI) has been reported inconsistently. The purpose of this study is to evaluate the effect of the catheterization timing and contrast media dose on the incidence of postoperative acute kidney injury.

**Methods:**

Patients who underwent cardiac catheterization and cardiac surgery successively from January 2015 to December 2015 were prospectively enrolled in this study. The primary outcome was CSA-AKI which was defined as the Kidney Disease: Improving Global Outcomes Definition and Staging (KDIGO) criteria. Univariate analysis and multivariate regression were performed to identify the predictors for CSA-AKI. Baseline characteristics were balanced with propensity score method for better adjustment.

**Results:**

A total of 1069 consecutive eligible patients were enrolled into this study. The incidence of CSA-AKI and AKI requiring renal replacement therapy (AKI-RRT) were 38.5% (412/1069) and 1.9% (20/1069) respectively. Preoperative estimated glomerular filtration rate less than 60 mL/min/1.73m^2^ (OR = 2.843 95% CI 1.374–5.882), the time interval between catheterization and surgery≤7 days (OR = 2.546, 95% CI 1.548–4.189) and the dose of contrast media (CM) > 240 mg/kg (OR = 2.490, 95%CI 1.392–4.457) were identified as predictors for CSA-AKI. In the patients with the dose of CM > 240 mg/kg, the incidence of CSA-AKI was higher in patients who underwent cardiac catheterization ≤7 days before cardiac surgery than in those of > 7 days before cardiac surgery (39.4% vs. 28.8%, *p* = 0.025). The longer interval of more than 7 days was revealed to be inversely associated with CSA-AKI through logistic regression (OR = 0.579, 95% CI 0.337–0.994).

**Conclusion:**

Catheterization within 7 days of cardiac surgery and a dose of CM > 240 mg/kg were associated with the onset of CSA-AKI. For patients who received a dose of CM > 240 mg/kg, postponing the cardiac surgery is potentially beneficial to reduce the risk of CSA-AKI.

## Background

Acute kidney injury (AKI) is one of the prevalent complications after cardiac surgery with reported incidences over 30% and associated higher mortality and excessive medical resource consumption [1–5]. Since no effective therapy is available to reverse the mechanism of AKI, early identification and modification of risk factors are necessary to prevent AKI [2].

Cardiac catheterization is one of the common pre-operative examinations. This procedure is aimed to screen the patients with high risk of coronary artery disease and provide clinicians with multiple cardiac surgical procedure alternatives. Recently, the association between pre-operative cardiac catheterization and postoperative acute kidney injury has been reported in several studies [6–11]. However, few of these studies interrogate both the interval between catheterization and surgery and the dose of contrast media (CM) simultaneously [11]. Moreover, these studies reported conflicting findings.

Accordingly, the purpose of this study is to identify the association between timing of catheterization, the dose of contrast media and postoperative AKI. The hypothesis is a shorter interval between catheterization and surgery and a higher dose of CM used in the catheterization are associated with increased incidence of AKI after surgery.

## Methods

### Study population

This study was based on the Zhongshan Cardiac Surgery Database, and the requirement for informed consent was waived by the ethical board from Zhongshan Hospital given the retrospective design. Patients who underwent cardiac catheterization and valve/ coronary artery bypass grafting (CABG)/valve + CABG at Fudan University Zhongshan Hospital in 2015 were included in this study. Patients with prior dialysis or underwent catheterization in other hospital and referred for surgery were excluded. Those who underwent urgent or salvage surgery were excluded as well. A dataset of 1069 eligible patients formed the study population. In case that more than one cardiac surgical procedure performed during the same hospitalization, only the data on the first surgery was considered. The datasets used and/or analysed during the current study are available from the corresponding author on reasonable request.

### Catheterization protocol

All patients in the dataset received iso-osmolar CM (Iodixanol, Visipaque®, GE Healthcare, Ireland) at iodine concentration of 320 mg/ml. The amount of CM was expressed in terms of milligrams per kilogram (mg/kg) to account for the iodine dose in different patients. Known nephrotoxic medications are discontinued 24 h before catheterization. Besides, adequate hydration of intravenous normal saline before angiography (1 ml/kg·h for 12 h), limiting of CM volume and intravenous sodium bicarbonate after angiography are routinely executed as standard protocol.

### Data definitions and endpoints

The interval time between the catheterization and the surgery was defined (days). Operation on the same day of the angiography was coded as interval 0. The cutoff value (7 days) of the interval derived from previous study [12] suggesting that, after being exposed contrast, renal function usually returns to preexisting levels within 7 days. The cutoff value for high contrast dose was determined with the receiving operating curve that had the maximal sum of sensitivity and specificity. In this study, it was found to be 240 mg/kg.

The primary endpoint was the occurrence of cardiac surgery associated acute kidney injury (CSA-AKI), which was defined as Kidney Disease: Improving Global Outcomes Definition and Staging (KDIGO) criteria [13] as any of the following: increase in serum creatinine (SCr) by ≥0.3 mg/dL (≥26.5 μmol/L) within 48 h; or increase in SCr to ≥1.5 times baseline that is known or presumed to have occurred within the prior 7 days or urine volume < 0.5 mL/kg per hour for 6 h. AKI was classified as KDIGO criteria: grade 1: post-op serum creatinine 1.5–1.9 times baseline or an increase in serum creatinine ≥26.5 μmol/L or a urine output < 0.5 ml/kg/h for 6–12 h; grade 2: post-op serum creatinine 2.0–2.9 times baseline or a urine output < 0.5 ml/kg/h for ≥12 h; and grade 3: post-op serum creatinine 3 times baseline or increase in serum creatinine to ≥353.6 μmol/L or initiation of renal replacement therapy or a urine output < 0.3 ml/kg/h for ≥24 h OR anuria for ≥12 h. The indications for renal replacement therapy (RRT) were metabolic abnormalities (acidosis, hyperkalemia), anuria, and fluid overload. The secondary endpoint was the in-hospital mortality.

Other perioperative variables in the present study included gender, age, comorbidities, preoperative cardiac function condition stratified with New York Heart Association (NYHA) classification, baseline serum creatinine and estimated glomerular filtration rate (eGFR) calculated with CKD-EPI formulae, procedures, cardiopulmonary bypass (CPB) duration (coded zero in patients operated without CPB) and erythrocyte transfusion, provided that they were used during surgery or the following 24 h. All data were checked twice by professional personnel before being admitted to the dataset.

### Statistics analysis

Continuous variables were expressed as the mean ± standard deviation (SD) and analyzed by unpaired t-tests, with Welch adjustment when necessary. Continuous variables that violated the normality assumption were expressed as median (interquartile range) and analyzed by a Mann-Whitney U test. Categorical variables were expressed as numbers (n) and percentage (%) and were analyzed by the Pearsonχ^2^ test or the Fisher exact test whenever appropriate.

Univariate analysis was performed to identify variables associated with CSA-AKI and those with *P* < 0.05 were included in the multivariate regression analysis to identify the independent risk factors for CSA-AKI. Propensity scores were later used as a way for better adjustment of the preoperative differences and control for selection bias. An adjusted logistic regression model was developed with variables that showed a *P* value < 0.05 in the univariate analysis. Significant level was considered with *P* < 0.05. Statistical analyses were performed by SPSS statistics for Windows (Version 24.0. IBM Corp, Armonk, NY).

## Results

### Baseline clinical and catheterization characteristics

Of 1069 eligible patients studied, 412 (38.5%) developed CSA-AKI according to KDIGO criterion. Among the AKI patients, the incidence of stage 2 AKI was 12.9% (53/412) and 6.1% (25/412) for stage 3 AKI. Renal replacement therapies were performed in 20 (1.9%) patients. Patients were stratified by the interval between catheterization and surgery into two groups, and their baseline characteristics are shown in Table 1. Most of the patients (83.1%) received surgery within 7 days. Compared with patients who underwent surgery after > 7 days, those undergoing surgery within 7 days were younger (61.3 ± 8.1 vs. 63.9 ± 8.3 years, *p* < 0.001) and were less frequently male (60.6% vs. 71.3%, *p* < 0.001), comorbid fewer hypertension (41.0% vs. 63.0%, *p* < 0.001) and diabetes mellitus (13.1% vs.30.9,% *p* < 0.001) but more NYHA 3–4 (77.3% vs. 65.2%, *p* < 0.001). The hemoglobin, albumin, serum creatinine and eGFR were similar. There were no significant differences in the amount of CM dose used in both cohorts. More valve surgery (77.9% vs.22.1%, *p* < 0.001) and CPB (79.4% vs.31.5%, *p* < 0.001) whereas less CABG (17.0% vs.72.4%, *p* < 0.001) were performed in the shorter interval cohort.

**Table 1:** Patients Perioperative Characteristics Stratified by the Interval between Coronary Angiography and Cardiac Surgery

	≤7 days (*N* = 888)	>7 days (*N* = 181)	*P*
Demographic data			
Male	538(60.6)	129(71.3)	0.007
Age (year)	61.3 ± 8.1	63.9 ± 8.3	< 0.001
Medical history			
Hypertension	364(41.0)	114(63.0)	< 0.001
DM	116(13.1)	56(30.9)	< 0.001
NYHA classification 3–4	686(77.3)	118(65.2)	< 0.001
Laboratory values			
Hemoglobin (g/L)	131.5 ± 15.1	130.9 ± 141.3	0.685
Albumin(g/L)	40.2 ± 3.5	39.8 ± 3.7	0.184
Kidney function			
Serum creatinine (mg/dl)	81.9 ± 24.5	85.8 ± 25.0	0.055
eGFR (ml/min/1.73m^2^)	83.8 ± 19.5	81.8 ± 20.8	0.227
eGFR< 60 ml/min/1.73m^2^	80(9.1)	24(13.9)	0.069
Angiographic values			
Interval between angiography and surgery (day)	3.2(1.9)	9.7(2.7)	< 0.001
^a^Contrast media dose (ml/kg)	280(231,349)	285(246,342)	0.198
Surgical data			
Valve	692(77.2)	40(22.1)	< 0.001
CABG	151(17.0)	131(72.4)	< 0.001
Valve & CABG	45(5.1)	10(5.5)	0.326
CPB	705(79.4)	57(31.5)	< 0.001
^a^CPB time (min)	100(75,127)	90(70,119)	0.106
^b^Erythrocyte transfusion	441(49.6)	75(41.4)	0.149
Prognosis			
AKI	374(42.1)	38(21.0)	< 0.001
KDIGO 1	305(34.3)	29(16.0)	< 0.001
KDIGO 2	45(5.1)	8(4.5)	0.286
KDIGO 3	24(2.7)	1(0.6)	< 0.001
RRT	16(1.8)	4(2.2)	0.762
In-hospital mortality	2(0.2)	1(0.6)	0.427
^a^Length of ICU stay (day)	40(22,64)	42(22,70)	0.861
^a^Length of hospital stay (day)	14(11,18)	12(11,15)	< 0.001

*AKI* Acute kidney injury, *DM* diabetes mellitus, *NYHA* New York Heart Association, *eGFR* estimated glomerular filtration rate, calculated by CKD-EPI formulae, *CABG* coronary artery bypass grafting, *CPB* cardiopulmonary bypass, *KDIGO* kidney disease: improving global outcomes, *RRT* renal replacement therapy, *ICU* intensive care unit

*P*-values are the results of unpaired t-test or Mann–Whitney U test for continuous variables, and × 2 test or Fisher’s exact test for categorical variables

^a^The values are expressed as the median (IQR)

^b^ The amount of erythrocyte transfusion refers to the amount of transfusion during both intraoperative and postoperative on the day of surgery

Concerning the endpoints, the incidence of AKI in the shorter interval cohort was twofold higher than the longer interval cohort (42.1% vs. 21.0%, p < 0.001) whereas the incidence of AKI requiring renal replacement therapy (AKI-RRT) and in-hospital mortality were similar. The intensive care unit (ICU) stay was similar while the hospital stay in the shorter interval cohort was longer than the longer interval cohort (14 vs. 12 days, *p* < 0.001).

### Univariate analysis

Univariate analysis was performed to identify variables associated with CSA-AKI (Table 2). Male gender, advanced NYHA classification, higher preoperative serum creatinine, more frequently valve surgery and less CABG performed, more frequently CPB and erythrocyte transfusion performed, and longer CPB duration was associated with the occurrence of CSA-AKI. Especially, eGFR less than 60 ml/min/1.73m^2^, shorter interval between catheterization and surgery and CM dose> 240 ml/kg increased CSA-AKI as well. Regarding secondary endpoint, mortality in the AKI patients was higher than those without AKI (0.7% vs. 0, *p* = 0.029). The length of ICU (47 vs. 32 h, *p* < 0.001) and hospital stay (13 vs. 12 days, p < 0.001) in the AKI patients were higher than those without AKI.

**Table 2:** Perioperative univariate analysis of AKI

	No AKI (*N* = 657)	AKI (*N* = 412)	*P*
Demographic data			
Male	394(60.0)	273(66.3)	0.039
Age (year)	61.8 ± 8.1	61.7 ± 8.3	0.936
Medical history			
Hypertension	294(44.7)	184(44.7)	0.977
DM	102(15.5)	70(17.0)	0.526
NYHA classification 3–4	465(70.8)	339(82.3)	< 0.001
Laboratory values			
Hemoglobin (g/L)	131.6 ± 14.2	131.1 ± 16.1	0.555
Albumin(g/L)	40.3 ± 3.6	40.0 ± 3.6	0.187
Kidney function			
Serum creatinine (mg/dl)	81.3 ± 25.1	84.5 ± 23.8	0.038
eGFR (ml/min/1.73m^2^)	83.9 ± 18.7	82.8 ± 21.3	0.395
eGFR< 60 ml/min/1.73m^2^	54(8.4)	50(12.1)	0.048
Angiographic values			
Interval between angiography and surgery (day)	4.5 ± 3.3	4.1 ± 3.0	0.048
^a^Contrast media dose (ml/kg)	285(243,341)	280(238,347)	0.696
Contrast media dose> 240 (ml/kg)	434(66.1)	302(73.3)	0.040
Surgical data			
Valve	427(65.0)	305(74.0)	< 0.001
CABG	205(31.2)	77(18.7)	< 0.001
Valve & CABG	25(3.8)	30(7.3)	0.026
CPB utilization	443(67.3)	319(77.4)	< 0.001
^a^CPB time (min)	83(67,109)	100(77,132)	< 0.001
^b^Erythrocyte transfusion	296(44.6)	223(54.1)	0.04
Prognosis			
RRT	3(0.5)	17(4.1)	< 0.001
In-hospital mortality	0	3(0.7)	0.029
^a^Length of ICU stay (day)	32(21,63)	47(26,90)	< 0.001
^a^Length of hospital stay (day)	12(10,15)	13(11,17)	0.003

*AKI* acute kidney injury, *DM* diabetes mellitus, *NYHA* New York Heart Association, *eGFR* estimated glomerular filtration rate, calculated by CKD-EPI formulae, *CABG* coronary artery bypass grafting, *CPB* cardiopulmonary bypass, *RRT* renal replacement therapy, *ICU* intensive care unit

*P*-values are the results of unpaired t-test or Mann–Whitney U test for continuous variables, and ×2 test or Fisher’s exact test for categorical variables

^a^The values are expressed as the median (IQR)

^b^The amount of erythrocyte transfusion refers to the amount of transfusion during both intraoperative and postoperative on the day of surgery

### Multivariate analysis

Multivariate analysis was performed to identify risk factors associated with CSA-AKI from the pool of variables that showed a *P* value < 0.05 in the univariate analysis (Table 3). Male gender (odds ration [OR] = 1.615, 95% 1.223–2.132), advanced NYHA classification (3–4) (OR = 1.683, 95% 1.225–2.311), combined surgery (OR = 2.595, 95% CI 1.376–4.892), eGFR less than 60 ml/min/1.73m^2^ (OR = 1.654, 95% CI 1.076–2.542), shorter interval between catheterization and surgery(OR = 2.184, 95%1.416–3.368) and CM dose> 240 ml/kg (OR = 1.346, 95%1.013–1.788) were found to be independent risk factors for CSA-AKI.

**Table 3:** Multivariate regression of risk factors for CSA-AKI

		Unadjusted			Adjusted	
	OR	95% CI	*P* value	OR	95% CI	*P* value
Male	1.615	1.223–2.132	0.001	1.568	0.871–2.821	0.133
Interval ≤ 7d	2.184	1.416–3.368	< 0.001	2.546	1.548–4.189	< 0.001
Contrast used> 240 mg/kg	1.346	1.013–1.788	0.041	2.490	1.392–4.457	0.002
NYHA 3–4	1.683	1.225–2.311	0.001	1.689	0.971–2.938	0.063
Valve & CABG	2.595	1.376–4.892	0.003	2.825	0.781–10.223	0.114
eGFR< 60 ml/min/1.73m^2^	1.654	1.076–2.542	0.022	2.843	1.374–5.882	0.005

*CSA-AKI* cardiac surgery associated acute kidney injury, *OR* odds ration, *CI* confidence interval, *NYHA* New York Heart Association, *CABG* coronary artery bypass grafting, *eGFR* estimated glomerular filtration rate, calculated by CKD-EPI formulae

Both Interval ≤ 7d and Contrast used> 240 mg/kg are revealed as predictors in the unadjusted and adjusted logistic regression analysis

Propensity score method was applied to control the selection bias related to covariates and better adjust the preoperative differences, and an adjusted logistic regression model was developed with variables that showed a P value < 0.05 in the univariate analysis. Preoperative estimated glomerular filtration rate less than 60 mL/min (adjusted OR = 2.843 95% CI 1.374–5.882), the time interval between catheterization and surgery≤7 days (adjusted OR = 2.546, 95% CI 1.548–4.189) and dose of CM > 240 mg/kg (adjusted OR = 2.490, 95% CI 1.392–4.457) were identified as predictors for CSA-AKI (Table 3).

### Subgroup analysis

The subgroup analysis was performed to elucidate the impact that shorter interval had on the development of CSA-AKI in the subjects exposed to higher dose CM (> 240 ml/kg). The incidence of CSA-AKI was higher in shorter interval patients than those had a longer interval (39.4% vs. 28.8%, *p* = 0.025). The longer interval of more than 7 days was revealed to be inversely associated with CSA-AKI (OR = 0.579, 95% CI 0.337–0.994) (Table 4).

**Table 4:** Multivariate analysis for CSA-AKI in the patients who underwent angiography with contrast used > 240 mg/kg

	OR	CI	*P* value
Interval > 7d	0.579	0.337–0.994	0.048
eGFR< 60 ml/min/1.73 m2	2.637	1.186–5.864	0.017

*CSA-AKI* cardiac surgery associated acute kidney injury, *OR* odds ration, *CI* confidence interval, *eGFR* estimated glomerular filtration rate, calculated by CKD-EPI formulae

Subgroup analysis revealed the interval between angiography and surgery > 7 days is negatively associated with CSA-AKI

In the subgroup of patients underwent on-pump surgery, 41.9% (319/762) developed AKI. Logistic regression showed male gender (OR = 1.763, 95% CI 1.293–2.404), shorter interval (≤7d) (OR = 2.427, 95% CI 1.270–4.637) and NYHA classification 3–4 (OR = 1.768, 95% CI 1.187–2.631) were found associated with the development of CSA-AKI (Table 5).

**Table 5:** Multivariate analysis for CSA-AKI in the patients who underwent on-pump surgery

	OR	95% CI	*P* value
Male gender	1.763	1.293–2.404	< 0.001
Interval ≤ 7d	2.427	1.270–4.637	0.007
NYHA 3–4	1.768	1.187–2.631	0.005

*CSA-AKI* cardiac surgery associated acute kidney injury, *OR* odds ration, *CI* confidence interval, *NYHA* New York heart association classification

Subgroup analysis revealed shorter interval between angiography and surgery (≤7d) is associated with CSA-AKI

## Discussion

The main findings of current study are (1) AKI after catheterization and cardiac surgery is prevalent, according to KDIGO definition, with the incidence of 38.5%; (2) the mortality of patients with AKI is significantly higher than those without AKI; (3) lower baseline eGFR, shorter interval between catheterization and cardiac surgery(< 7 days) and higher CM dose(> 240 mg/kg) used are associated with the development of CSA-AKI; (4) in the patients receiving higher dose of CM, postponing cardiac surgery is possibly protective for reducing AKI risk.

Postoperative acute kidney injury remains one of the major complications with worse prognosis [3, 5]. Effective prevention is predominantly dependent on the early identification of risk factors. Many risk factors have been revealed associating with CSA-AKI [1]. However, few of them are modifiable, but their clinical implications are significant.

The American College of Cardiology/American Heart Association guidelines for the management of patients with valve heart disease has a class I level of recommendation to perform angiography in patients scheduled for valve surgery [14]. While catheterizations are prevalently performed before cardiac surgery to screen potential coronary disease and indicate appropriate surgery, the adverse effect CM have on the kidney, which is known as contrast induced nephropathy (CIN), requires concern.

Among multiple mechanisms contributing to CIN, renal ischemia, particularly in the medulla, reactive oxygen species formation, reduction of nitric oxide production, and tubular epithelial and vascular endothelial injury are reported frequently [15, 16]. Patients with pre-existing renal impairment with reduced nephron mass are susceptible to the influence of CM [17, 18]. The decline in kidney function usually occurs 1 to 3 days after the procedure. Renal function usually returns to pre-existing levels within 7 days [12]. Assuming that kidney was compromised by “double strike” from cardiac surgery associated adverse effect like hemodynamic instability and inflammatory reaction when kidney function has not recovered, the risk of CSA-AKI increases theoretically.

As modifiable features, the interval between catheterization and surgery as well as the CM dose and their association with CIN have been targeted for preventing AKI after surgery [6–8, 10, 11, 19, 20]. Conflicting data have been reported. Several authors proposed that closer succession between two procedures have an adverse impact on the development of CSA-AKI [6, 8, 10, 11], whereas other authors claimed the risk of CSA-AKI is not influenced by the time interval [7, 21]. The main differences across the studies are attributed to variant AKI definitions and different surgical procedures. A meta-analysis encompassing nine studies showed that the time interval of 1 day or less between CAG and on-pump cardiac surgery was significantly associated with increased risk of AKI [22]. Another recent study, in which AKI was defined as KDIGO criteria, showed that on-pump CABG performed within 7 days after catheterization was a predictor of postoperative AKI [19]. Consistent with this finding, a similar outcome resulted through the subgroup analysis in the present study. In the patients had CPB during their valve or CABG, catheterization within 7 days of surgery was a predictor for CSA-AKI.

The amount and type of CM used during catheterization have also been reported to affect the incidence of CIN as well. The nephrotoxic effect of iodinated CM may be proportional to dose. However, there is no evidence of a dose-toxicity relationship when administered at usual diagnostic dose [23]. Administration of higher CM volumes is associated with increased CIN rate in patients with chronic kidney disease or diabetes mellitus [24–26]. The osmolality and viscosity of the CM have been reported to participate in their renal toxicity [27, 28]. With routine angiography protocol (see Methods), the amount of CM used in the patients in our institute is limited. The CM used in present study population is iodixanol, a non-ionic iso-osmolar CM, which has been shown to have the lowest associated risk of CIN and mortality [29, 30]. However, in the present study, a higher dose of CM remained to be an independent predictor of CSA-AKI. In the subgroup consisted of patients received CM dose> 240 mg/kg, the prolonged interval before surgery was inversely associated with the development of CSA-AKI. This result has the clinical implication. A higher dose of CM are usually administrated in patients whose underlying heart disease are complex and more precise diagnosis and evaluation by high-quality angiography are needed. These patients can be more vulnerable to the successive strike of complicated surgery, which by itself increases the risk of AKI.

Based on the findings, the current study has clinical significance. Patients who underwent cardiac surgery within 7 days are at higher risk of developing CSA-AKI. Especially when the patient has moderate or worse preoperative kidney dysfunction or receiving a higher dose of CM during the catheterization, postponing cardiac surgery to more than 7 days will be potentially beneficial to prevent post-operative AKI.

The main limitation of this study is that it is an observational trial. The hypothesis that catheterization timing and CM dose are associated with CSA-AKI has been generated in this study, whereas the causality between them shall be drawn deliberately. In spite of a single center study, the propensity score was used to correct for selection bias and baseline difference. Moreover, the individualized dose of CM was expressed in terms of iodine concentration/patient body weight to make it comparable with other iodinate CM.

## Conclusion

The current study showed catheterization within 7 days of cardiac surgery, a dose of contrast media over 240 mg/kg and preoperative eGFR less than 60 ml/min/1.73m^2^ were associated with increased risk of CSA-AKI. The findings suggest that delaying elective cardiac surgery is potentially beneficial to prevent CSA-AKI, especially in patients with impaired preoperative kidney dysfunction or those receiving a higher dose of CM. It remained to be verified in the future whether these strategies, along with other protocol, can reduce CSA-AKI incidence and improve prognosis.

## Abbreviations

AKI: Acute kidney injury
CABG: Coronary artery bypass grafting
CM: Contrast media
CPB: Cardiopulmonary bypass
DM: Diabetes mellitus
eGFR: Estimated glomerular filtration rate, calculated by CKD-EPI formulae
ICU: Intensive care unit
KDIGO: Kidney Disease: Improving Global Outcomes
NYHA: New York Heart Association
RRT: Renal replacement therapy
